# A Novel Broadband Band-pass Filter Based on Spoof Surface Plasmon Polaritons

**DOI:** 10.1038/srep36069

**Published:** 2016-10-31

**Authors:** Lei Zhao, Xin Zhang, Jun Wang, Wenhua Yu, Jiandong Li, Hai Su, Xiaopeng Shen

**Affiliations:** 1 Center for Computational Science and Engineering, Jiangsu Normal University, China; 2 State Key Laboratory of Millimeter Waves, Southeast University, China; 3 Department of Physics, China University of Mining and Technology, China

## Abstract

In this paper, we present a novel broadband bandpass filter based on spoof surface plasmon polaritons (SSPPs) in the microwave frequency band. The proposed bandpass filter includes three parts: (1) coplanar waveguide (CPW); (2) matching transition; and (3) coupled structure that is an asymmetric coupled filter constructed by five grooved strips. The proposed bandpass filter realizes excellent low loss performance from 7 to 10 GHz, in which its insertion loss is around 1.5 dB in the same frequency band. Meanwhile, this filter has a good band stop characteristic from 3 to 7 GHz. A simple but accurate transmission line model was proposed to evaluate the proposed broadband SSPPs filter. The measured data, simulated results and the results obtained from the transmission line model have shown a very good agreement. The proposed planar broadband filter plays an important role for filtering surface plasmon polaritons (SPPs) waves in plasmonic circuits and systems.

Surface plasmon polaritons (SPPs) are highly localized surface waves in the optical frequency region, which propagate parallel to the interface between air and metal and decay exponentially in the vertical direction, since the metal has the similar property to plasma with a negative permittivity^1^,^2^. Many researches have demonstrated that the SPPs have the ability to confine light in a subwavelength scale with high intensity, which can be used to tackle the diffraction limit, miniaturize photonic components, and build highly integrated optical components and circuits^3^,^4^. The SPPs have attracted great attentions owing to their huge potential applications in the areas of surface characterization, biomedical sensing, near-field microscopy, nano-photonic and optoelectronic technologies^5^,^6^,^7^.

When the interested frequency is reduced to terahertz (THz) and microwave bands, the metals behave akin to perfectly electric conductors (PECs), and hence the natural SPPs cannot be invoked on the metal surface. Plasmonic metamaterials have been proposed to produce the so-called spoof SPPs (SSPPs)^8^,^9^,^10^,^11^,^12^,^13^,^14^,^15^, which can produce highly confined surface electromagnetic (EM) wave at the lower frequencies. An important advantage of this metamaterial is that the dispersion characteristics and spatial confinement of the SSPPs can be controlled by the geometrical parameters of array elements.

As the plasmonic metamaterials are advancing at a rapid pace, they are considered to be a promising candidate in the practical applications^16^,^17^,^18^,^19^,^20^,^21^,^22^. The broadband bandpass filters are key components and play a very important role in various communication and radar systems. A SPPs bandpass filter was designed using a bulky structure with a periodic subwavelength metallic Domino array^23^. Recently, efficient filtering effect and ultrathin spoof surface plasmonic waveguides were already reported^24^,^25^,^26^,^27^,^28^,^29^,^30^,^31^,^32^,^33^,^34^, which show excellent filtering characteristics such as low loss and wideband. However, the existing articles have not given a clear theoretical analysis on the designs. Thereafter, more efforts are still towards to design a bandpass filter with a wide stopband that is highly demanded in practical applications for effective suppression of the undesired noise signal.

In this paper, an ultrathin metallic structure printed on a thin flexible dielectric substrate is proposed to achieve a broadband bandpass filter with a wide bandpass in the microwave frequency band, which is composed of two asymmetrically broken corrugated strips coupled via the grooved strips with an embedded split ring. Two transition sections are also designed to obtain the smooth conversion between the CPW and the SSPPs filter, which can extract the transmitted SSPPs wave from the traditional CPW. The measurement of the SSPPs filter demonstrates a good agreement with the simulated results. Meanwhile, a simple but accurate transmission line model was first proposed to evaluate the transmission and reflection properties of the proposed filter. Using this compact structure, the proposed SSPPs filter with a low loss and good transmission in the pass band has the absolute advantages in the design of the plasmonic integrated circuits in both microwave and terahertz frequency bands.

## Results

**Figure 1:**
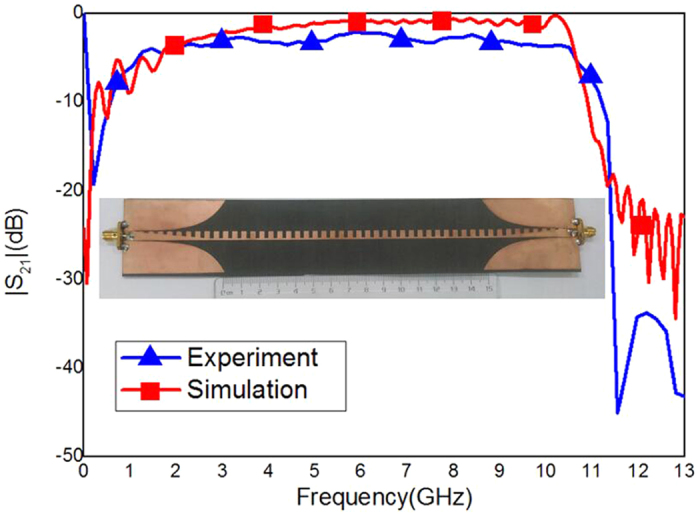
Simulated and measured |*S*_21_| of the SSPPs waveguide and the photograph of the fabricated SSPPs waveguide.

As a well-known fact, the SSPPs waveguide has an excellent transmission property, as shown in Fig. 1, whose performance is much better than the traditional microstrip^25^. Many related works based on this structure have been reported. For example, a planar composite plasmonic waveguide can achieve three or more signals transmission with a good propagation performance^26^. A broadband and high-efficiency transition is designed to connect a microstrip line to a conformal surface plasmon (CSP) waveguide^27^. The spoof plasmonic waveguide has much lower propagation loss and longer propagation length compared with the conventional spoof plasmonic waveguide with rectangular grooves^28^.

**Figure 2:**
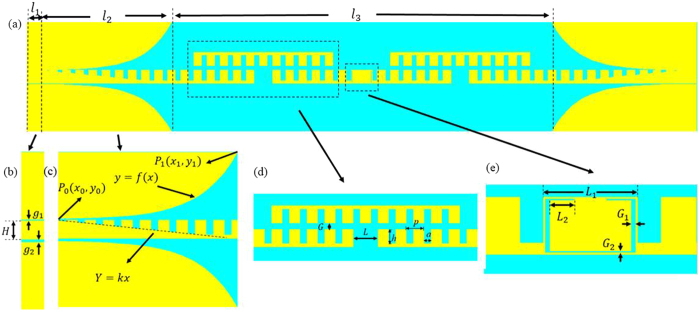
The configuration of SSPPs filter. (**a**) Top view of the proposed broadband filter, in which *l*_*1*_ = 5 *mm*, *l*_*2*_ = 50 *mm*, and *l*_*3*_ = 115 *mm*. (**b**) CPW structure, in which *g*_*1*_ = *0.13* *mm, g*_*2*_ = *0.26 mm and H* = *5 mm*. (**c**) Matching transition with the gradient grooves and flaring ground, in which *k* = *4/45*. (**d**) Zoomed view of the couple structure, in which *G* = 1.8 *mm, L* = 7 *mm, h* = 4 *mm, p* = 5 *mm and a* = 2 *mm*. (**e**) Zoomed view of the split ring, in which *L*_1_ = 8 *mm*, *L*_2_ = 2.1 *mm*, *G*_1_ = 0.4 *mm* and *G*_2_ = 0.15 *mm*.

**Figure 3:**
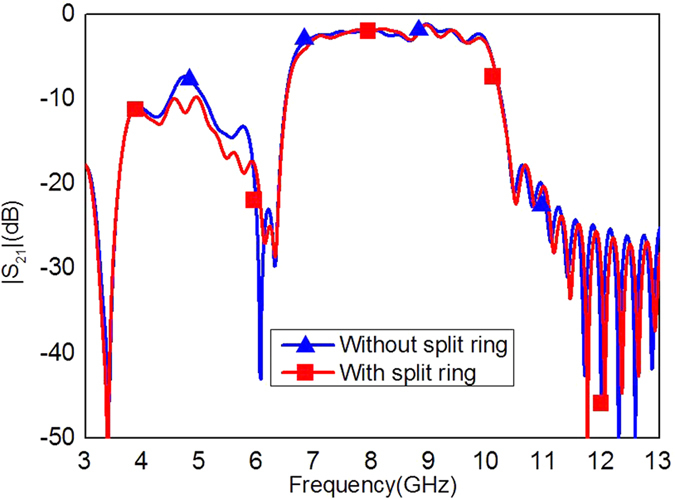
Simulated transmission coefficients |*S*_21_| with and without the split ring slot.

Based on this type of SSPPs waveguides, a broadband filter is proposed and optimized in this section, as shown in Fig. 2(a). The yellow and light blue parts in Fig. 2 stand for metal (copper) and flexible thin dielectric substrate, respectively. The thickness of the ultrathin metallic strips is selected to be 0.018 mm and the F4B substrate has a thickness of 0.5 mm. The corrugated metallic strip is launched via two CPWs with a 50 Ω characteristic impedance, and the parameters of this particular structure are accurately retrieved as: *l*_*1*_ = 5 *mm*, *l*_*2*_ = 50 *mm*, and *l*_*3*_ = 115 *mm*. The dash line in Fig. 2(c) is described by using the expression *Y* = *kx (k* = *4/45)*. The Vivaldi curve of flaring ground is expressed as:


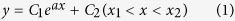


where 
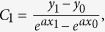
 
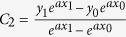
, *a = *0.08. *P*_*0*_(*x*_*0*_, *y*_*0*_), *P*_*1*_(*x*_*1*_, *y*_*1*_) in Fig. 2(c) are the coordinates of the start and end points of the Vivaldi^35^. The coupling structure of the proposed filter is illustrated in Fig. 2(d). The period *p*, the width *a*, and the depth *h* of metallic grooves are selected to be 5 *mm*, 2 *mm* and 4 *mm*, respectively; the gap *L* between the two lower strips is chosen to be 7 *mm*; and the gap *G* between the lower and upper strips is chosen to be 1.8 mm. A split ring slot in the middle element of the filter, as shown in Fig. 2(e), is designed to improve the reflection in the stop band. The dimensions of the split ring slot are optimized to be: the frame length *L*_1_ = 8 *mm*, the shoulder length of the inner patch *L*_2_ = 2.1 *mm*, the side gap *G*_1_ = 0.4 *mm*, and the bottom gap *G*_2_ = 0.15 *mm*. The simulated |*S*_21_| of the filter with and without the split ring slot are demonstrated in Fig. 3. It is observed from Fig. 3 that the split ring slot makes the |*S*_21_| less than −10 dB at 4.5 GHz.

**Figure 4:**
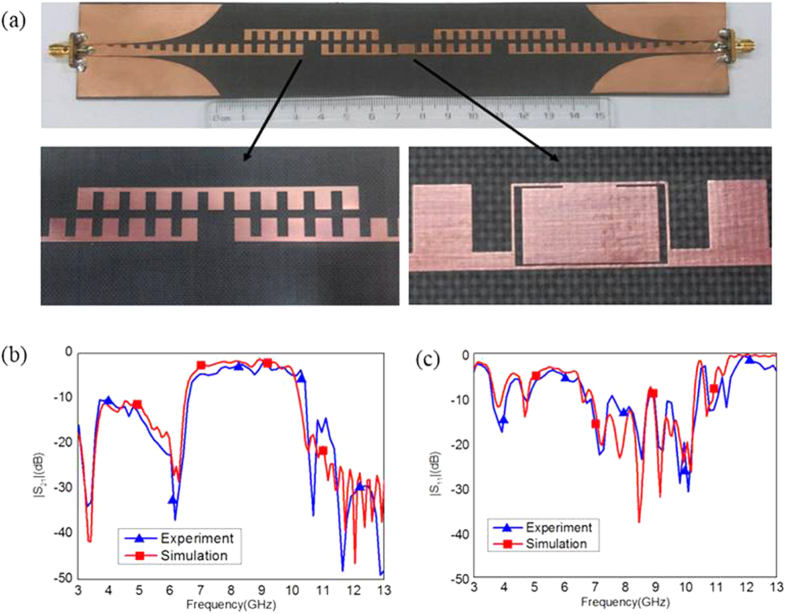
The photograph of the fabricated SSPPs filter and the simulation and measurement result of S-parameters. (**a**) Photograph of the fabricated SSPPs filter mounted on a flexible dielectric film. (**b**) The transmission coefficients. (**c**) The reflection coefficients.

In the microwave frequency band, the fabricated filter is shown in Fig. 4(a). The measured and simulated S-parameters (|*S*_11_| and |*S*_21_|) are plotted in Fig. 4(b,c). It is evident from Fig. 4 that there exists a good agreement between the simulated results and the measured data. We also notice from Fig. 4 that |*S*_11_| is less than −10 dB from 7 to 10 GHz, implying an excellent impedance match of the transition structure from the CPW to the SSPPs filter. It is a fact that the filter only has around 1.5 dB transmission loss in the pass band.

**Figure 5:**
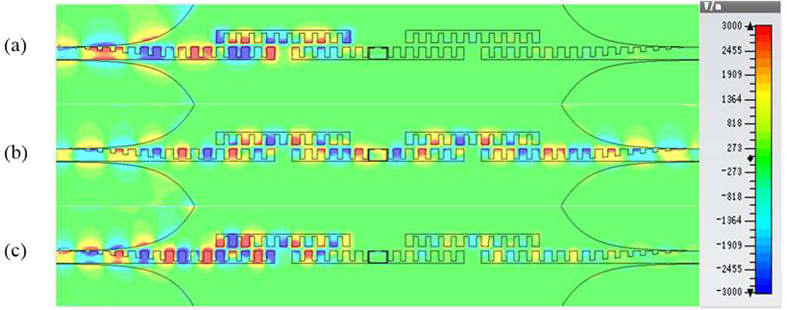
Simulated electric-field distributions on the element side of the proposed filter at different frequencies. (**a**) *f* = 6 GHz. (**b**) *f* = 8 GHz. (**c**) *f* = 10 GHz.

In order to gain insight into the band-pass characteristics of the proposed filter, we present the near-field distributions on the filter surface at different frequencies, as shown in Fig. 5. It is obvious from Fig. 5 that the wave energy cannot propagate through the coupling structure at 6 GHz and 11 GHz since such frequencies are outside the band-pass frequency band. The excellent propagation property at the operating frequency 8 GHz is clearly observed from Fig. 5(b).

### Transmission Line Model

**Figure 6:**
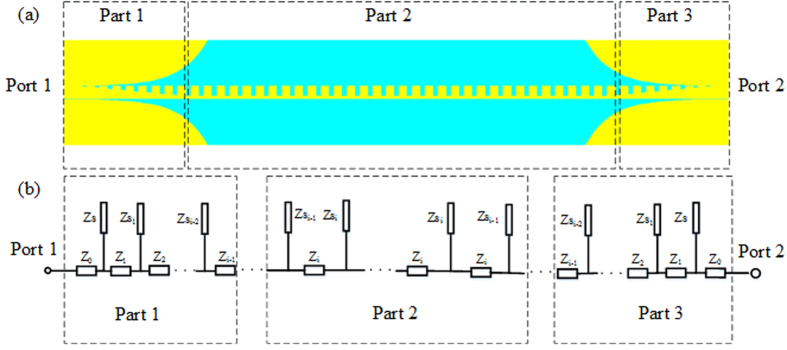
Schematic diagram of the proposed SSPPs waveguide. (**a**) Transmission line model of the SSPPs waveguide. (**b**) Equivalent circuit of the SSPPs waveguide.

The SSPPs waveguide includes a single conductor and seems different from the conventional microwave transmission line. Since the spoof waveguide transmits the wave by coupling between the grooves, its equivalent model can be analyzed by using the transmission line model^36^,^37^ by considering a very far ground plane at infinity. Based on this definition, we can calculate the capacitors which connect the line to the ground^36^. This model matches the conventional cases and one can thus use the impedance, scattering and ABCD matrix definitions. Such curved ground design has been used already in another application of using SSP to feed dielectric resonator antennas (DRAs) to achieve isotropic radiation^37^. In order to validate the proposed transmission line model, we first use it to evaluate the SSPPs waveguide, as shown in Fig. 6. Assuming that the vertical transmission lines have no coupling, the ABCD matrices of the short stub *M*_*i*_ and the open stub *N*_*i*_^38^ are expressed as:


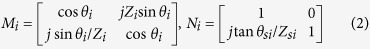


where *Z*_*i*_ and *Z*_*si*_ are characteristic impedances of the short and open stubs, respectively. *θ*_*i*_ and *θ*_*si*_ are the electrical lengths of the short and open stubs, respectively. The ABCD matrix of the entire circuit can be expressed as:


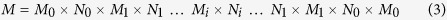


**Figure 7:**
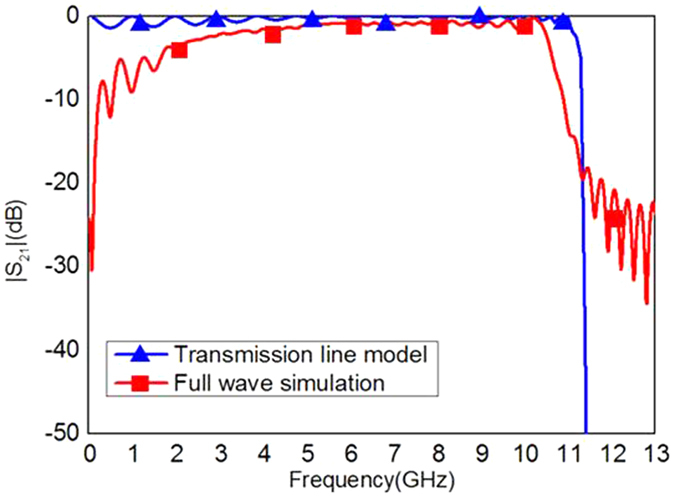
Comparison of |*S*_21_| of the proposed SSPPs waveguide obtained from the transmission line model and the full wave simulation.

Then, using the cascade connection theory of two-port network, *S*_11_ and *S*_21_ are obtained as:


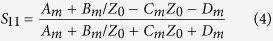



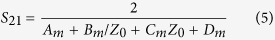


where *A*_*m*_, *B*_*m*_, *C*_*m*_, and *D*_*m*_ are the elements of matrix *M*. The comparison of |*S*_21_| obtained by using the full wave simulation and the transmission line model is demonstrated in Fig. 7, and it is evident that they are in a good agreement. The deviation at lower frequencies between the transmission line model and the full wave simulation is caused by the time domain solver used in this paper, which is not accurate at the low frequencies.

**Figure 8:**
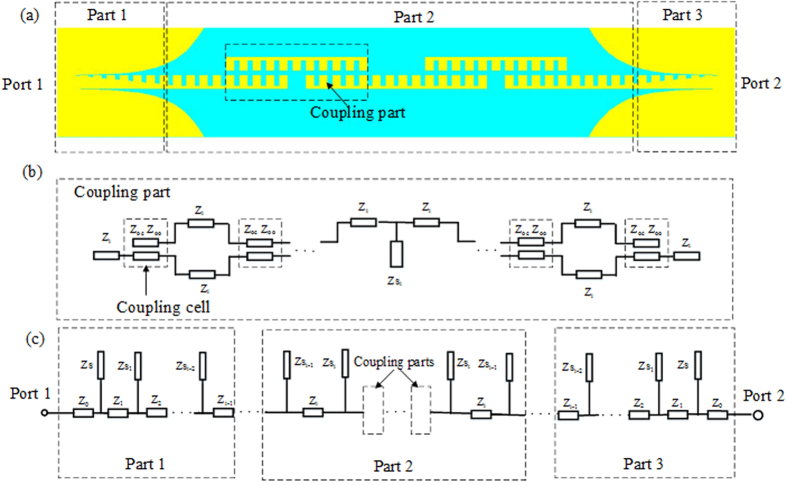
Schematic diagram of the proposed broadband band-pass filter based on the SSPPs waveguide. (**a**) Model of the broadband band-pass filter. (**b**) Coupling part of the broadband band-pass filter. (**c**) Equivalent circuit of the broadband band-pass filter.

**Figure 9:**
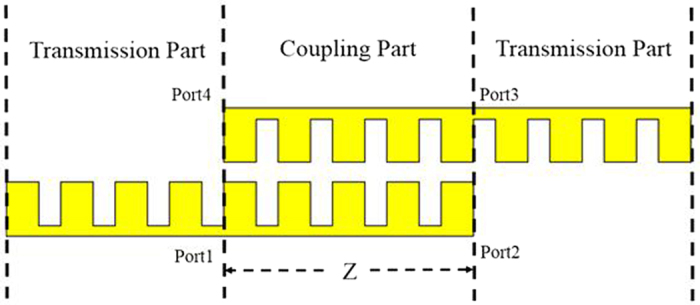
Schematic of the coupling part of the proposed filter.

The transmission line model for the proposed broadband filter includes the coupling part and equivalent circuit part, as shown in Fig. 8. The detailed structure for the coupling part is demonstrated in Fig. 8(b), in which *Z*_*oe*_ and *Z*_*oo*_ are the even- and odd-mode characteristic impedances of the coupling unit, respectively. Based on the concept of microwave network, the coupling part can be described by a four-port network^39^, as shown in Fig. 9. We can get the following relationships for the ports 1, 2, 3, and 4:


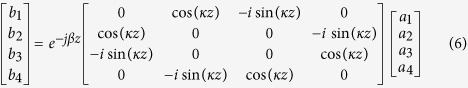



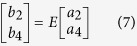


where *κ* is the coupling coefficient, and *z* is the length of coupling cell. The *S* scattering parameter matrix of the coupling part is


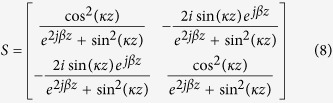


**Figure 10:**
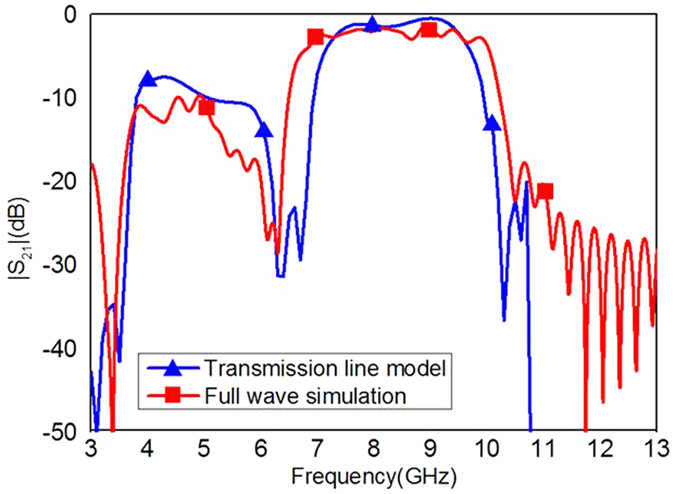
Transmission coefficients of the proposed SSPPs filter obtained by using the transmission line model and the full wave simulation.

On the base of the equivalent circuit^40^ and Eqs (2, 3, 4, 5, 6, 7), we can get the final *S*_11_ and *S*_21_. Figure 10 gives |*S*_21_| of the SSPPs filter obtained by using the full wave simulation and equivalent transmission line model, which shows a good agreement between the full wave simulated results and the ones obtained from transmission line model.

## Discussion

In this paper, we propose a novel bandpass filter constructed by an ultrathin metallic structure printed on a thin dielectric substrate to generate a broadband property in the microwave frequency band. The filter is composed of two broken corrugated strips and three coupled strips with an embedded ring slot. The traditional CPW is employed to excite the filter and extract the transmitted SSPPs parameters, and two transition sections are designed for smooth conversion between the CPW and the SSPPs waveguide. The performance of the proposed filter has been analyzed using the equivalent transmission line model. The measurement results show that the reflect coefficient is less than −10 dB with the transmission loss around 1.5 dB in the frequency band from 7 to 10 GHz. Such performance makes the proposed structure be a good filter, which may be further used in multilayer structures in the future for higher integrations.

## Methods

With the help of commercial software, CST Microwave Studio, we simulated the dispersion relations, S-parameters and surface fields of the waveguide and filter. As shown in Figs 1(a) and 4(a), the experimental structure is fabricated using a 0.5 *mm* thin dielectric film with dielectric constant 2.65 and loss tangent 0.001, respectively. The thickness of metal (copper) film is 0.018 *mm*. The Agilent Vector Network Analyzer (E5063A) was used to measure the S-parameters (i.e., the reflection coefficients *S*_11_ and transmission coefficients *S*_21_) of the fabricated samples.

## Additional Information

**How to cite this article**: Zhao, L. *et al*. A Novel Broadband Band-pass Filter Based on Spoof Surface Plasmon Polaritons. *Sci. Rep*. **6**, 36069; doi: 10.1038/srep36069 (2016).

**Publisher’s note:** Springer Nature remains neutral with regard to jurisdictional claims in published maps and institutional affiliations.
